# Survival after first relapse in marginal zone lymphomas: Prospective data from the international observational NF10 study

**DOI:** 10.1111/bjh.70808

**Published:** 2026-08-30

**Authors:** Stefano Luminari, Maria Elena Nizzoli, Michele Merli, Sara Rattotti, Vittoria Tarantino, Simone Ferrero, Annalisa Talami, Roberta Murru, Marina Deodato, Emanuele Cencini, Giovanni Roti, Carlo Visco, Guilherme Duffles, Michele Spina, Ombretta Annibali, Alessandro Pulsoni, Andrés José María Ferreri, Caterina Stelitano, Elsa Pennese, Emiliano Barbieri, Marzia Varettoni, Gambara Silvia, Marta Coscia, Dario Marino, Tisi Maria Chiara, Maher Nawar, Luigi Marcheselli, Francesco Merli, Luca Arcaini

**Affiliations:** ^1^ Division of Hematology Azienda Unità Sanitaria Locale IRCCS of Reggio Emilia Reggio Emilia Italy; ^2^ CHIMOMO Department University of Modena and Reggio Emilia Reggio Emilia Italy; ^3^ Division of Hematology Fondazione IRCCS Ca' Granda, Ospedale Maggiore Policlinico Milan Italy; ^4^ Division of Hematology Fondazione IRCCS Policlinico San Matteo and University of Pavia Pavia Italy; ^5^ Division of Hematology Azienda Ospedaliera Ospedali Riuniti Villa Sofia‐Cervello Palermo Italy; ^6^ Hematology Division, Department of Molecular Biotechnology and Health Sciences University of Torino Torino Italy; ^7^ Clinical and Experimental Medicine Doctorate School Università Degli Studi di Modena e Reggio Emilia Reggio Emilia Italy; ^8^ Hematology and Stem Cell Transplantation Unit Ospedale Oncologico A. Businco, ARNAS G. Brotzu Cagliari Italy; ^9^ Department of Hematology Niguarda Cancer Center, ASST Grande Ospedale Metropolitano Niguarda Milan Italy; ^10^ Division of Hematology Azienda Ospedaliera Universitaria Senese & University of Siena Siena Italy; ^11^ Division of Hematology Azienda Ospedaliera Universitaria di Parma Parma Italy; ^12^ Hematology and Bone Marrow Transplant Unit, Department of Engineering for Innovative Medicine University of Verona Verona Italy; ^13^ Hematology and Hemotherapy Centre, University of Campinas Sao Paulo Brazil; ^14^ Division of Medical Oncology IRCCS Centro Di Riferimento Oncologico Aviano Italy; ^15^ Division of Hematology, Stem Cell Transplantation Fondazione Policlinico Universitario Campus Bio Medico Rome Italy; ^16^ Division of Hematology, Department of Translational and Precision Medicine, S.M. Goretti Hospital Sapienza University—Polo Pontino Latina Italy; ^17^ Strategic Program on Lymphomas IRCCS San Raffaele Scientific Institute, Milano and Vita‐Salute San Raffaele University Milan Italy; ^18^ Division of Hematology Grande Ospedale Metropolitano, Bianchi Melacrino Morelli Reggio Calabria Italy; ^19^ Centro Diagnosi e Terapia Dei Linfomi, Presidio Ospedaliero Spirito Santo Pescara Italy; ^20^ Division of Hematology University of Insubria and ASST Sette Laghi, Ospedale di Circolo of Varese Varese Italy; ^21^ Oncology IOV Padua Italy; ^22^ Hematology Ospedale San Bortolo Vicenza Italy; ^23^ Hematology UNIUPO Novara Italy; ^24^ Fondazione Italiana Linfomi, Clinical Trial Office Modena Italy; ^25^ Department of Molecular Medicine University of Pavia Pavia Italy

**Keywords:** haematology, marginal zone lymphoma, MZL‐IPI, NF10 study, progression‐free survival from first relapse

## Abstract

While marginal zone lymphoma (MZL) is generally indolent, outcomes following relapse remain poorly defined. This study utilized the indolent Non Follicular 10 (NF10) prospective database to characterize survival and identify prognostic drivers in patients failing initial systemic therapy. The study analysed 122 patients with relapsed MZL. The primary end‐point was progression‐free survival from first relapse (2PFS), with survival after relapse (SAR) as the secondary end‐point. The median 2PFS was 24 months, with a 2‐year 2PFS rate of 49% and a 2‐year SAR rate of 66%. Multivariable analysis identified advanced age, progression of disease within 24 months (POD24) and high MZL International Prognostic Index (MZL‐IPI) scores as significant predictors of inferior survival. Patients with splenic and disseminated subtypes also demonstrated worse outcomes. Relapse following systemic therapy often marks a transition to an aggressive clinical course. Time to progression and the MZL‐IPI are robust tools for risk stratification, highlighting an urgent need for novel therapeutic strategies for high‐risk relapsed MZL populations.

## INTRODUCTION

Marginal zone lymphomas (MZL) represent a heterogeneous group of indolent B‐cell lymphomas, comprising three main subtypes: splenic (SMZL), nodal (NMZL) and extra‐nodal MZL (ENMZL), also known as mucosa‐associated lymphoid tissue (MALT) lymphoma.^1^, ^2^ These entities share a generally favourable prognosis following front‐line therapy, often characterized by indolent clinical behaviour, responsiveness to immunochemotherapy and prolonged survival. Nonetheless, relapse is a common event in MZL, particularly in patients with disseminated or high‐risk disease features.^3^, ^4^, ^5^, ^6^

While therapeutic strategies in the front‐line setting are relatively well defined, ranging from watch‐and‐wait in asymptomatic cases to rituximab‐based regimens in active disease, the optimal management of relapsed or refractory MZL is less established. Treatment choices at relapse are often heterogeneous and guided by patient characteristics, prior therapies and institutional preferences rather than standardized guidelines. Importantly, clinical trials in the relapsed setting have frequently included small and heterogeneous MZL subpopulations, limiting generalizability. Moreover, few studies have provided prospective, real‐world data on outcomes after relapse, and prognostic factors in this setting remain underexplored.

Several retrospective studies have suggested that biological features such as the MZL International Prognostic Index (MZL‐IPI),^4^ early progression after treatment (POD24)^3^, ^7^ and elevated lactic dehydrogenase (LDH) may influence post‐relapse outcomes. However, these findings require validation in prospective cohorts. Additionally, the prognostic impact of MZL subtype at relapse, in particular for SMZL and disseminated forms, has not been comprehensively addressed in prospective studies.

The NF10 registry, performed under the sponsorship of the Fondazione Italiana Linfomi (FIL), offers a unique opportunity to address these gaps by collecting prospective data on indolent non‐follicular lymphomas, including detailed information on clinical course, treatment and outcomes.^3^, ^4^

Within this context, we performed a focused analysis on MZL patients experiencing their first relapse after systemic therapy. The primary objective was to describe post‐relapse outcomes in terms of progression‐free survival from relapse (2PFS) and survival after relapse (SAR). Secondary objectives included the identification of prognostic factors associated with adverse outcomes, with the aim of informing risk‐adapted strategies and identifying unmet needs in this setting.

## METHODS

The NF10 registry (NCT02904577) prospectively enrolled patients with indolent non‐follicular B‐cell lymphomas across 65 centres in 8 countries of Europe and South America (2010–2018). All patients had histologically confirmed disease and were managed per institutional protocols.

Selection criteria for the present study were MZL (NMZL, SMZL and EMZL) and adults who experienced relapse after first‐line chemotherapy and/or immunotherapy. The date of first relapse was defined as the index date. Patient characteristics at index date were collected and reported as baseline characteristics. Details on the treatment administered immediately before and after index date were collected.

End‐points of this analysis are 2PFS, defined as the time from date of first relapse to subsequent relapse, progression or death by any cause and SAR, defined as the time from date of first relapse to death from any cause. Patients alive at last clinical control were censored.

### Statistical analysis

Baseline features were summarized using descriptive statistics, and group comparisons were performed using Fisher's exact test or chi‐square test.

2PFS and SAR were estimated using the Kaplan–Meier method with confidence interval at 95% (95% CI). Associations with outcomes were tested using univariable and multivariable Cox proportional hazard (PH) regression models and the effect of covariate was estimated by hazard ratio (HR) with 95% CI. All p‐values were two‐sided.

## RESULTS

Out of 790 MZL patients enrolled in the NF10 registry, systemic therapy was administered in 435 immediately after diagnosis while 97 patients were treated with local therapy. Among the remaining 258 patients who were initially addressed to active surveillance, 66 required systemic therapy due to progression to symptomatic disease. Overall, 501 patients received systemic therapy and 122 experienced a first relapse, thus representing our study population (Figure S1 and Table S1). MZL subtypes were as follows: SMZL (36%), NMZL (15%), ENMZL (29%) and disseminated MZL (20%) (Table 1). Median time from initial diagnosis to first relapse was 21 months (range 1–82) and was 15, 37, 20 and 22 months in SMZL, NMZL, ENMZL and disseminated MZL respectively.

**TABLE 1:** Characteristics of 122 patients with marginal zone lymphoma progressed/relapsed after first treatment at time of progression/relapse.

Covariate	Status	*n*/*N*	%
Age at progression	>70	62/122	51
Time to first progression	<12 months	34/122	28
POD24	Not achieved	69/122	57
LDH	>ULN	47/103	46
β_2_‐microglobulin	>ULN	52/78	67
Platelets	<1 × 10^9^/L	19/100	19
Haemoglobin	<12 g/dL	58/101	57
Absolute lymphocyte count (ALC)	<1 × 10^9^/L	39/95	41
Histology	ENMZL	35/122	29
	SMZL	44/122	36
	NMZL	18/122	15
	Diss. MZL	25/122	20
MZL‐IPI at progression	Low	13/95	14
	Intermediate	48/95	51
	High	34/95	36

*Note*: POD24 not achieved: progression of disease within 24 months.

At index date, 51% had age >70 years, 46% had elevated LDH; according to the MZL‐IPI (1), 14% were low risk, 51% intermediate and 36% high risk on 95 evaluable patients while 57% were classified as POD24 (Table 1). Details about second‐line treatments were available in 91 patients (57%) and included rituximab in 70% of cases; autologous haematopoietic stem cell transplant (AHSCT) was performed in 12%. Looking at risk transition according to MZL‐IPI calculated at baseline and at index date, approximately half of baseline low risk were reclassified at intermediate/high risk when they relapsed while baseline intermediate/high risk cases maintained the same profile at time of progression (Table S2).

Median follow‐up from index date was 31 months (range: 1–84). Median 2PFS was 24 months (95% CI: 14–54); 2‐year 2PFS rate was 49% (95% CI: 39%–58%) (Figure 1A). 2PFS was shorter in patients aged >70 years, POD24 and high MZL‐IPI (HR high vs. low: 6.58; 95% CI: 1.97–21.9) as reported in Table 2. The 2‐year 2PFS for IPI‐MZL at progression was 100%, 55% (95% CI 39–68) and 40% (95% CI 23–57) for low, intermediate and high score respectively (log rank *p* = 0.002) (Figure 2).

**FIGURE 1:**
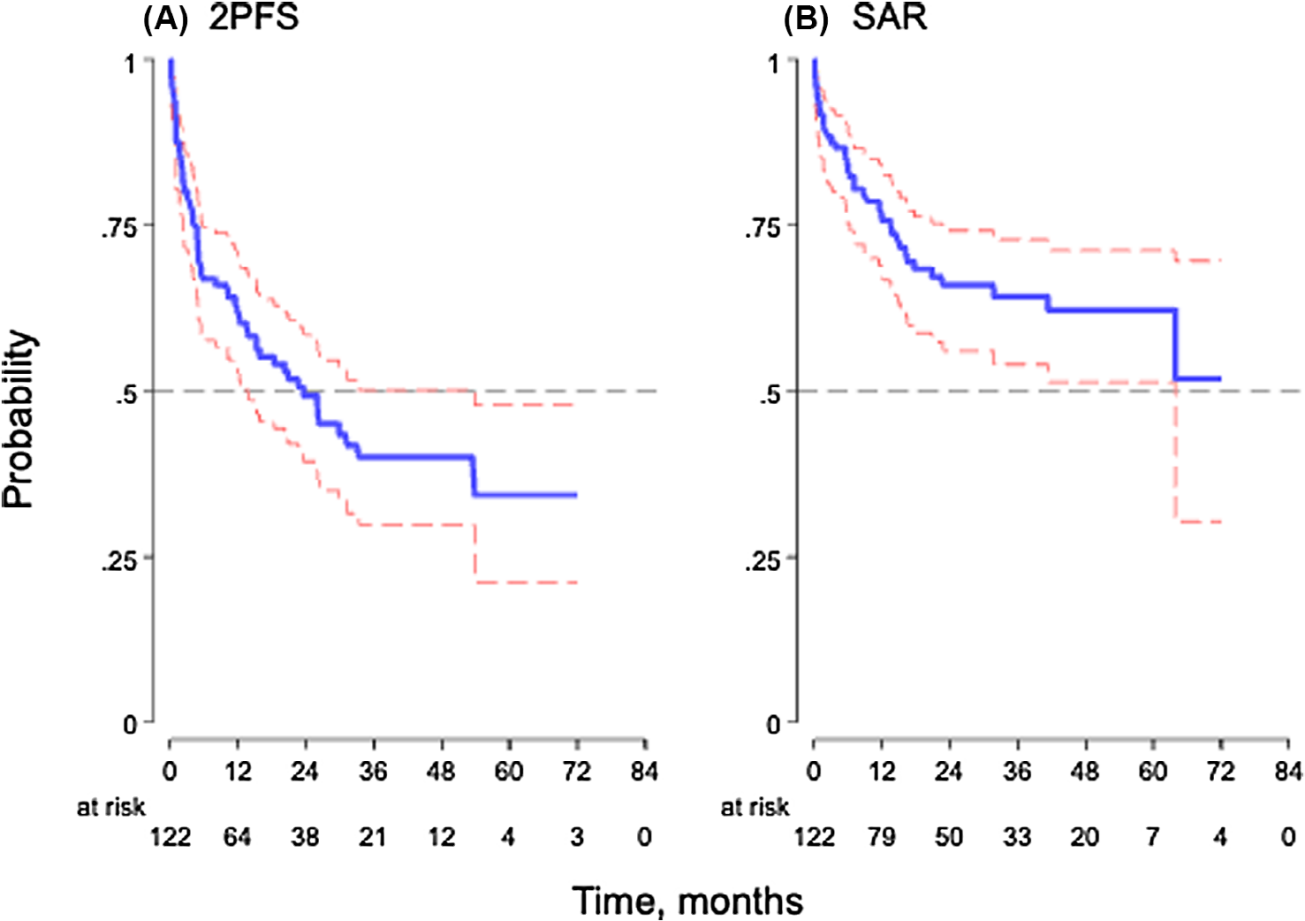
Kaplan–Meier curves for 2PFS (A) and SAR (B) of 122 patients with MZL r/r after first treatment at time.

**TABLE 2:** Univariable Cox PH regression for progression‐free survival from index date (2PFS).

Factor	HR (95% CI)	*p*
Treatment after W&W	1.52 (0.72–3.21)	0.266
Age at index date >70 years	1.75 (1.05–2.89)	0.030
Male sex	0.94 (0.57–1.54)	0.792
IPI‐MZL (at index date)		
Low	1.00	
Intermediate	3.21 (0.98–10.5)	0.053
High	6.58 (1.97–21.9)	0.002
First treatment without Rituximab	1.65 (0.88–3.10)	0.122
POD24 not achieved	1.97 (1.15–3.39)	0.014
Time to first progression <12 months	1.29 (0.76–2.20)	0.353
Salvage treatments		
R‐Bendamustine	1.00	
Treatment with Rituximab	2.36 (1.15–4.82)	0.019
Treatment without Rituximab	2.62 (1.17–5.86)	0.019
AHSCT	1.38 (0.52–3.64)	0.514
Not available	2.17 (1.03–4.58)	0.042
MZL subtype		
ENMZL	1.00	
SMZL	3.03 (1.50–6.14)	0.002
NMZL	2.05 (0.82–5.13)	0.127
Disseminated MZL	2.88 (1.33–6.23)	0.007
LDH > ULN [*n* = 101]	1.60 (0.90–2.81)	0.048
β_2_‐microglobulin > ULN [*n* = 78]	2.18 (1.00–4.76)	0.050
Haemoglobin < 12 mg/dL [*n* = 101]	4.49 (2.15–9.38)	<0.001
Platelets < 100 × 10^9^/L [*n* = 100]	3.35 (1.77–6.35)	<0.001
ALC <1 × 10^9^/L [*n* = 95]	1.81 (0.99–3.30)	0.052

*Note*: [*n*=] cases available at progression/relapse; POD24 not achieved: Progression of disease within 24 months.

Abbreviation: AHSCT, autologous haematopoietic stem cell transplant.

**FIGURE 2:**
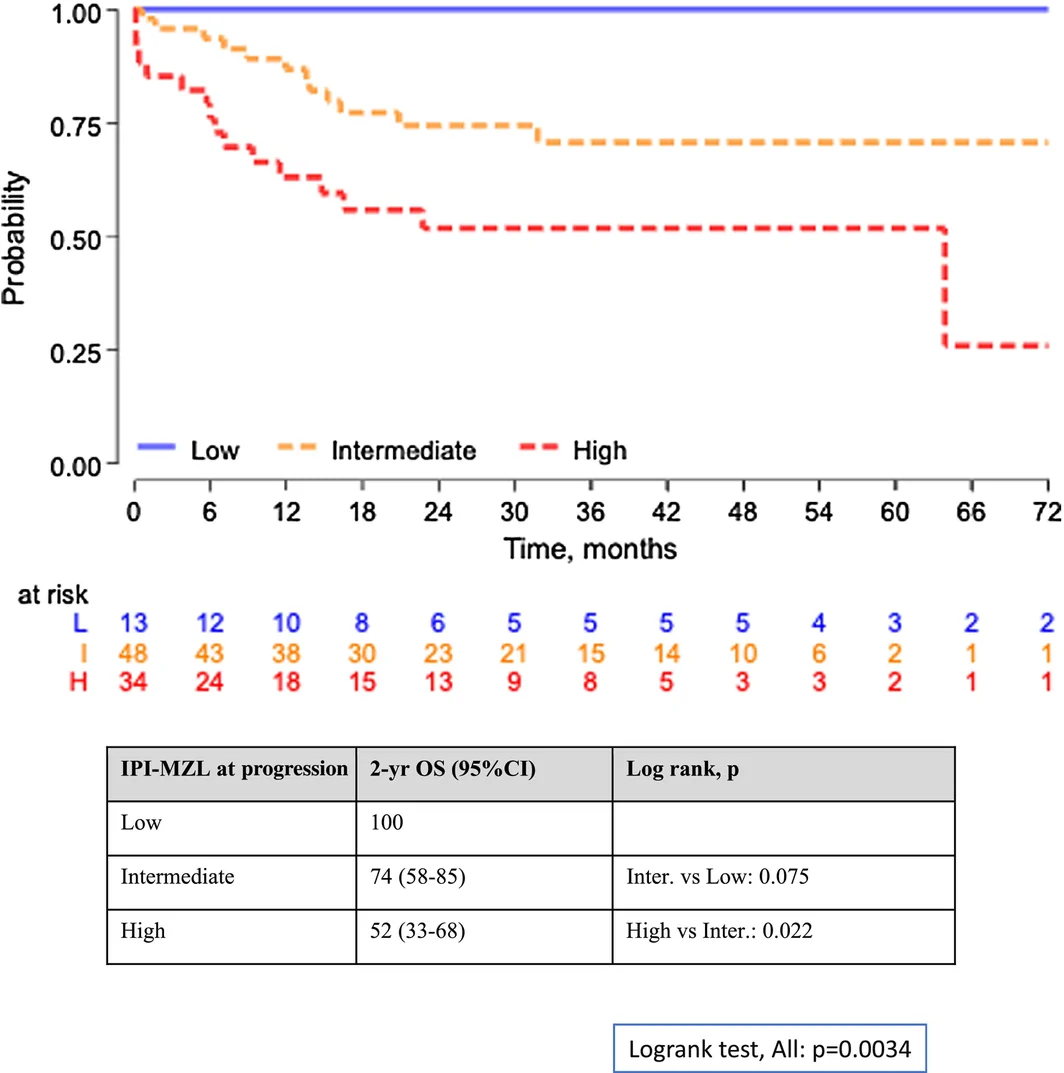
Progression‐free survival from index date (2PFS) according to MZL‐IPI at relapse.

Median SAR was not reached, and 2‐year SAR was 66% (95% CI: 56–74) (Figure 1B). SAR was adversely impacted by age >70 years, POD24, high MZL‐IPI, SMZL and disseminated MZL subtypes. Prior watch‐and‐wait (Table 3) or local therapy (data not shown) did not affect outcomes. The 2‐year SAR for IPI‐MZL at progression was 100%, 74% (95% CI 58–85), and 52% (95% CI 33–68) for low, intermediate and high score respectively (log rank *p* = 0.003) (Figure 2). (PFS and overall survival [OS] survival by baseline MZL‐IPI is reported in Figure S2).

**TABLE 3:** Univariable Cox PH regression for survival after relapse (SAR) in 122 patients with marginal zone lymphoma progressed/relapsed after first treatment.

Factor	HR (95% CI)	*p*
Treatment after W&W	1.65 (0.69–3.93)	0.258
Age at progression >70 years	2.06 (1.08–3.92)	0.028
Male sex	0.77 (0.41–1.44)	0.412
IPI‐MZL (at treatment)		
Low	1.00	
Intermediate	3.99 (0.53–30.2)	0.181
High	17.4 (2.33–129)	0.005
First treatment without Rituximab	1.96 (0.93–4.12)	0.076
POD24 not achieved	3.81 (1.68–8.63)	0.001
Time to first progression <12 months	1.40 (0.73–2.68)	0.315
Salvage treatments		
R‐Bendamustine	1.00	
Treatment with Rituximab	2.11 (0.84–5.24)	0.109
Treatment without Rituximab	3.94 (1.55–10.0)	0.004
AHSCT	1.23 (0.33–4.64)	0.762
Not available	1.87 (0.70–5.03)	0.211
MZL subtype		
ENMZL	1.00	
SMZL	3.03 (1.50–6.14)	0.002
NMZL	2.05 (0.82–5.13)	0.127
Disseminated MZL	2.88 (1.33–6.23)	0.007
LDH > ULN [*n* = 101]	1.44 (0.73–2.82)	0.291
β_2_‐microglobulin > ULN [*n* = 78]	1.95 (0.72–5.24)	0.188
Haemoglobin <12 mg/dL [*n* = 101]	14.2 (3.38–59.3)	<0.001
Platelets <100 × 10^9^/L [*n* = 100]	4.41 (2.11–9.21)	<0.001
ALC <1 × 10^9^/L [*n* = 95]	1.73 (0.82–3.64)	0.148

*Note*: [*n*=] cases available at progression/relapse. POD24 not achieved: Progression of disease within 24 months.

Abbreviation: AHSCT, autologous haematopoietic stem cell transplant.

The POD after first progression was available in 75 patients who received a systemic salvage therapy: among these patients, 37 (49%) had a second progression of disease within 24 months from index date; the 2‐year SAR by POD after first‐ or second‐line therapy was 96% and 48%, respectively (log rank *p* < 0.001) (Figures S3 and S4).

## DISCUSSION

This prospective analysis from the NF10 registry provides one of the most comprehensive real‐world evaluations of outcomes of patients with MZL relapsed after a first line of systemic therapy: the median 2PFS of 24 months and a 2‐year SAR of 66% underscore that MZL, while generally considered an indolent condition, may follow a more aggressive course, in particular if relapse occurs early or is associated with high‐risk features.

Consistent with previous evidence, our study validates the prognostic relevance of POD24, age >70 years and high‐risk MZL‐IPI in the relapse setting.^3^, ^4^ Similar to what was observed in follicular lymphomas, however, effective second‐line therapies^8^ were able to correct the expected poor outcomes associated with early relapse to first‐line therapy (POD24).

Notably, the strong prognostic impact of MZL‐IPI at relapse suggests its potential utility beyond the frontline context, offering a standardized tool for post‐relapse risk stratification. These findings may assist in clinical decision‐making, helping identify patients who may benefit from more aggressive salvage strategies or enrolment in clinical trials.

A particularly novel and relevant aspect of our analysis is the differential outcome across MZL subtypes: SMZL and disseminated forms had significantly worse outcomes both in terms of 2PFS and SAR, reinforcing the need for subtype‐specific therapeutic approaches. Indeed, while ENMZL is often characterized by localized disease and favourable response to radiotherapy or antibiotics (in specific settings), SMZL and disseminated MZL often present with higher tumour burden, bone marrow involvement and cytopenias, factors potentially contributing to inferior survival outcomes. These findings further support the inclusion of MZL subtype in future prognostic models.

Interestingly, prior watch‐and‐wait strategy or local therapy did not negatively impact outcomes after relapse, suggesting that deferring treatment in asymptomatic patients remains a reasonable approach; this is particularly important in the context of indolent lymphomas where overtreatment can be avoided without compromising long‐term prognosis.

From a therapeutic standpoint, the heterogeneity of second‐line treatments reported in our cohort reflects the current lack of standardized approaches in relapsed MZL. The relatively limited use of AHSCT (12%) and the observation that both rituximab‐containing and rituximab‐free regimens were used to highlight an area of clinical uncertainty. Emerging agents such as Bruton tyrosine kinase (BTK) inhibitors (e.g. ibrutinib,^9^ zanubrutinib^10^) and lenalidomide‐based combinations have shown promising efficacy in relapsed/refractory MZL,^11^ particularly in patients with limited options. However, these agents were not routinely used in our cohort due to the enrolment period (2010–2018) and regional regulatory approvals.

Another important issue is the role of markers such as LDH, β2‐microglobulin and cytopenias at relapse, which were associated with inferior outcomes; while these markers are not specific to MZL, their routine availability and prognostic value support their integration into clinical risk models.

Our study has limitations mainly due to the small sample size and to the high clinical heterogeneity of MZL patients: treatment strategies were not standardized and reflect real‐world practice, introducing potential heterogeneity. Molecular and genetic data were not systematically collected, precluding the evaluation of novel biomarkers such as *NOTCH2*, *KLF2* or *MYD88* mutations,^12^ which may further refine prognostication in the future. Finally, the relatively small number of events in some subgroups limits the precision of estimates, particularly in multivariable models.

In conclusion, with the limitations of a small study, this prospective analysis shows that survival after relapse in MZL is heterogeneous and influenced by well‐established clinical factors as well as disease subtype. When confirmed in larger studies, these findings suggest the adoption of risk‐adapted strategies at relapse, the need for international consensus on salvage treatment algorithms, and further exploration of novel agents in high‐risk populations. The integration of molecular profiling and standardized prognostic indices such as MZL‐IPI may enhance personalized management in the relapsed setting. Availability of effective novel therapies in relapsed refractory MZL is eagerly awaited.

## AUTHOR CONTRIBUTIONS

**Vittoria Tarantino:** Data curation; investigation; writing – original draft; writing – review and editing. **Michele Merli:** Data curation; investigation; writing – original draft; writing – review and editing. **Sara Rattotti:** Data curation; investigation; writing – original draft; writing – review and editing. **Emanuele Cencini:** Investigation; data curation; writing – original draft; writing – review and editing. **Marina Deodato:** Data curation; investigation; writing – review and editing; writing – original draft. **Annalisa Talami:** Data curation; investigation; writing – review and editing; writing – original draft. **Stefano Luminari:** Conceptualization; data curation; investigation; formal analysis; writing – original draft; writing – review and editing. **Maria Elena Nizzoli:** Data curation; investigation; writing – original draft; writing – review and editing. **Giovanni Roti:** Data curation; investigation; writing – review and editing; writing – original draft. **Guilherme Duffles:** Data curation; investigation; writing – review and editing; writing – original draft. **Alessandro Pulsoni:** Data curation; investigation; writing – original draft; writing – review and editing. **Marzia Varettoni:** Data curation; investigation; writing – original draft; writing – review and editing. **Dario Marino:** Data curation; investigation; writing – original draft; writing – review and editing. **Carlo Visco:** Data curation; investigation; writing – original draft; writing – review and editing. **Simone Ferrero:** Investigation; data curation; writing – original draft; writing – review and editing. **Elsa Pennese:** Data curation; investigation; writing – review and editing; writing – original draft. **Roberta Murru:** Data curation; investigation; writing – original draft; writing – review and editing. **Andrés José María Ferreri:** Data curation; investigation; writing – review and editing; writing – original draft. **Marta Coscia:** Data curation; investigation; writing – review and editing; writing – original draft. **Tisi Maria Chiara:** Investigation; data curation; writing – review and editing; writing – original draft. **Luca Arcaini:** Conceptualization; formal analysis; writing – original draft; writing – review and editing; investigation; data curation. **Gambara Silvia:** Data curation; investigation; writing – review and editing; writing – original draft. **Maher Nawar:** Investigation; data curation; writing – review and editing; writing – original draft. **Emiliano Barbieri:** Data curation; investigation; writing – review and editing; writing – original draft. **Michele Spina:** Data curation; investigation; writing – original draft; writing – review and editing. **Luigi Marcheselli:** Data curation; investigation; formal analysis; writing – original draft; writing – review and editing. **Caterina Stelitano:** Data curation; investigation; writing – original draft; writing – review and editing. **Francesco Merli:** Investigation; data curation; writing – review and editing; writing – original draft. **Ombretta Annibali:** Data curation; investigation; writing – review and editing; writing – original draft.

## FUNDING INFORMATION

Funding was provided by Fondazione Italiana Linfomi.

## CONFLICT OF INTEREST STATEMENT

The authors report no conflicts of interest in this work.

## ETHICS STATEMENT

Study protocol is approved by the Ethics Committee of the participating centres. Informed consent was obtained from all subjects involved in the study. The research was conducted in accordance with the Declaration of Helsinki.

## Supporting information


**Table S1.** Baseline characteristics of 122 patients with marginal zone lymphoma progressed/relapsed after first treatment.
**Table S2.** Probability transition matrix MZL‐IPI from baseline to progression (*n* = 93).
**Figure S1.** Flow chart of the study.
**Figure S2.** 2PFS and SAR stratified by baseline MZLIPI (*N* = 117).
**Figure S3.** SAR stratified by POD24 after first progression.
**Figure S4.** Survival after relapse (SAR) by POD24 after first‐ and second‐line therapy.

## Data Availability

Data are available upon request.
