# Supplementary material for: Survival after first relapse in marginal zone lymphomas: Prospective data from the international observational NF10 study
Source: Br J Haematol. 2026 Aug 30;209(5):2066–72. doi: 10.1111/bjh.70808 (PMC13748388; doi:10.1111/bjh.70808)
Supplement: Supplementary file 1 [file BJH-209-2066-s001.docx]

**Supplementary material**

**Supplementary Table 1** - Baseline characteristics of 122 patients with marginal zone lymphoma progressed/relapsed after first treatment

| **Covariate** | **Status** | **n/N** | **%** |
| --- | --- | --- | --- |
| **Age at treatment** | >70 years | 58/122 | 48 |
| **Sex** | Male | 58/122 | 47 |
| **Treatment 1st line** | At diagnosis | 110 | 90 |
|  | From W&W | 12 | 10 |
| **MZL subtype** | ENMZL | 35 | 29 |
|  | SMZL | 44 | 36 |
|  | NMZL | 18 | 15 |
|  | Disseminated MZL | 25 | 20 |
| **Treatment 1st line** | With Rituximab | 100/122 | 82 |
| **LDH** | > ULN | 49/119 | 41 |
| **β_2_-microglobulin** | > ULN | 73/98 | 74 |
| **Platelets** | < 100 x10^9^/L | 23/120 | 19 |
| **Hemoglobin** | < 12 g/dL | 65/120 | 54 |
| **ALC** | < 1 x 10^9^/L | 33/119 | 28 |
| **MZL-IPI** | Low | 16/117 | 14 |
|  | Intermediate | 67/117 | 57 |
|  | High | 34/117 | 29 |

LDH, Lactic Dehydrogenase; ALC, Absolute Lymphocyte counts; UNL, Upper Normal Limit; SMZL, Splenic Marginal Zone Lymphoma; ENMZL. Extranodal Marginal zone Lymphoma; NMZL, Nodal Marginal Zone Lymphoma; Diss, Disseminated; ENS: # extranodal sites; ALC: absolute lymphocyte count; IPI-MZL: international prognostic index of MZL.

Details of first line therapies

| Type | N (%) |
| --- | --- |
| Mono alkylating agent | 14 (11.5) |
| R-monotherapy | 13 (10.7) |
| R-alkylant | 29 (23.8) |
| R-CHOP | 19 (15.6) |
| R-Bendamustine | 39 (32.0) |
| R-fludarabine | 4 (2.3) |
| Other | 3.3 |
| Total | 122 |

**Supplementary Table 2** - Probability transition matrix MZL-IPI from baseline to progression (n=93)

| **MZL-IPI** | **Progression** | | |
| --- | --- | --- | --- |
| Baseline | 0 | 1-2 | 3-5 |
| 0 | 46.1 | 53.8 | 0 |
| 1-2 | 11.5 | 63.5 | 25.0 |
| 3-5 | 3.6 | 28.6 | 67.9 |

**Supplementary Figure 1** - Flow chart of the study

**Supplementary Figure 2** - 2PFS and SAR stratified by baseline MZLIPI (N=117)

| MZL-IPI | 3-yr 2PFS (95%CI) | Logrank, p | 3-yr SAR (95%CI) | Logrank, p |
| --- | --- | --- | --- | --- |
| Low | 81 (51-93) |  | 93 (61-99) |  |
| Intermediate | 39 (25-53) | I vs L: 0.035 | 77 (63-86) | I vs L: 0.147 |
| High | 25 (12-42) | H vs I: 0.006 | 52 (33-68) | H vs I: <0.001 |

**Supplementary Figure 3** - SAR stratified by POD24 after first progression.

Achieved	3-yr SAR 100%

Not achieved	3-yr SAR 44% (95%CI 26-60%)	logrank, p <0.001

|  | **POD after second line.** | |  |
| --- | --- | --- | --- |
| **POD24 after first line** | **>24** | **≤24** | **Total** |
| >24 | 15 (39%) | 14 (38%) | 29 (39%) |
| ≤24 | 23 (61%) | 23 (62%) | 46 (61%) |
| **Total (% by row)** | 38 (51%) | 37 (49%) | 75 |

Correlation between POD24 after first and second line of therapy

**Supplementary Figure 4** - Survival after relapse (SAR) by POD24 after first and second line therapy

| **SAR (adjusted according Casulo eta al.)** | | | |  |
| --- | --- | --- | --- | --- |
| POD 1st & POD post | **n (%)** | **2-yr % (95%CI)** | **Logrank** | **p** |
| a) POD post >24 | 38 (51) | 96 (74-99) | b vs a | 0.019 |
| b) POD 1st>24 & post <24 | 14 (19) | 67 (34-87) | c vs a | <0.001 |
| c) POD 1st/post both <24 | 23 (31) | 39 (19-58) | c vs b | 0.100 |
